# Supplementary material for: Role and mechanism of NCAPD3 in promoting malignant behaviors in gastric cancer
Source: Front Pharmacol. 2024 Apr 22;15:1341039. doi: 10.3389/fphar.2024.1341039 (PMC11070777; doi:10.3389/fphar.2024.1341039)
Supplement: Supplementary file 11 [file DataSheet2.ZIP › GSEA/Oncogenic signature/my_analysis.Gsea.1599461962066/BMI1_DN_MEL18_DN.V1_UP.html]

Details for gene set BMI1\_DN\_MEL18\_DN.V1\_UP[GSEA]

|  || Dataset | filtered\_dataset.sample\_info.cls#WT\_versus\_NCAPD3\_MUT |
| Phenotype | sample\_info.cls#WT\_versus\_NCAPD3\_MUT |
| Upregulated in class | NCAPD3\_MUT |
| GeneSet | BMI1\_DN\_MEL18\_DN.V1\_UP |
| Enrichment Score (ES) | -0.46890223 |
| Normalized Enrichment Score (NES) | -2.4435627 |
| Nominal p-value | 0.0 |
| FDR q-value | 0.0027402148 |
| FWER p-Value | 0.004 |
Table: GSEA Results Summary

  

Fig 1: Enrichment plot: BMI1\_DN\_MEL18\_DN.V1\_UP      
 Profile of the Running ES Score & Positions of GeneSet Members on the Rank Ordered List

  

| SYMBOL | TITLE | RANK IN GENE LIST | RANK METRIC SCORE | RUNNING ES | CORE ENRICHMENT || 1 | 323 | APBB2 | 35 | 0.977 | 0.0376 | No |
| 2 | 6573 | SLC19A1 | 231 | 0.650 | -0.0615 | No |
| 3 | 4297 | KMT2A | 337 | 0.573 | -0.1005 | No |
| 4 | 2919 | CXCL1 | 405 | 0.534 | -0.1146 | No |
| 5 | 5292 | PIM1 | 885 | -0.300 | -0.4416 | No |
| 6 | 8140 | SLC7A5 | 890 | -0.305 | -0.4248 | No |
| 7 | 5329 | PLAUR | 952 | -0.373 | -0.4449 | Yes |
| 8 | 10123 | ARL4C | 957 | -0.377 | -0.4235 | Yes |
| 9 | 7286 | TUFT1 | 1010 | -0.412 | -0.4346 | Yes |
| 10 | 3914 | LAMB3 | 1015 | -0.414 | -0.4108 | Yes |
| 11 | 10938 | EHD1 | 1019 | -0.419 | -0.3860 | Yes |
| 12 | 23046 | KIF21B | 1034 | -0.433 | -0.3683 | Yes |
| 13 | 51200 | CPA4 | 1035 | -0.434 | -0.3403 | Yes |
| 14 | 8609 | KLF7 | 1071 | -0.457 | -0.3362 | Yes |
| 15 | 9076 | CLDN1 | 1084 | -0.463 | -0.3150 | Yes |
| 16 | 11221 | DUSP10 | 1095 | -0.475 | -0.2917 | Yes |
| 17 | 7204 | TRIO | 1120 | -0.494 | -0.2772 | Yes |
| 18 | 2152 | F3 | 1188 | -0.559 | -0.2896 | Yes |
| 19 | 23657 | SLC7A11 | 1205 | -0.581 | -0.2638 | Yes |
| 20 | 29968 | PSAT1 | 1215 | -0.589 | -0.2324 | Yes |
| 21 | 8061 | FOSL1 | 1222 | -0.597 | -0.1982 | Yes |
| 22 | 330 | BIRC3 | 1224 | -0.600 | -0.1604 | Yes |
| 23 | 57761 | TRIB3 | 1292 | -0.685 | -0.1647 | Yes |
| 24 | 8744 | TNFSF9 | 1305 | -0.708 | -0.1278 | Yes |
| 25 | 1839 | HBEGF | 1328 | -0.751 | -0.0953 | Yes |
| 26 | 6509 | SLC1A4 | 1330 | -0.752 | -0.0476 | Yes |
| 27 | 7422 | VEGFA | 1346 | -0.788 | -0.0077 | Yes |
| 28 | 115123 | MARCH3 | 1360 | -0.828 | 0.0362 | Yes |
Table: GSEA details [plain text format]

  

Fig 2: BMI1\_DN\_MEL18\_DN.V1\_UP      
 Blue-Pink O' Gram in the Space of the Analyzed GeneSet

  

Fig 3: BMI1\_DN\_MEL18\_DN.V1\_UP: Random ES distribution      
 Gene set null distribution of ES for **BMI1\_DN\_MEL18\_DN.V1\_UP**
